# Stock Market Forecasting Based on Spatiotemporal Deep Learning

**DOI:** 10.3390/e25091326

**Published:** 2023-09-12

**Authors:** Yung-Chen Li, Hsiao-Yun Huang, Nan-Ping Yang, Yi-Hung Kung

**Affiliations:** 1 Department of Statistics and Information Science, Fu Jen Catholic University, New Taipei City 242062, Taiwan; yuki050151@gmail.com (Y.-C.L.); stat2021@mail.fju.edu.tw (H.-Y.H.); 2 Department of Mathematics, Fu Jen Catholic University, New Taipei City 242062, Taiwan; yang@math.fju.edu.tw

**Keywords:** stock forecasting, spatiotemporal transformer, spacetimeformer model, multi-steps forecasting

## Abstract

This study introduces the Spacetimeformer model, a novel approach for predicting stock prices, leveraging the Transformer architecture with a time–space mechanism to capture both spatial and temporal interactions among stocks. Traditional Long–Short Term Memory (LSTM) and recent Transformer models lack the ability to directly incorporate spatial information, making the Spacetimeformer model a valuable addition to stock price prediction. This article uses the ten minute stock prices of the constituent stocks of the Taiwan 50 Index and the intraday data of individual stock on the Taiwan Stock Exchange. By training the Timespaceformer model with multi-time-step stock price data, we can predict the stock prices at every ten minute interval within the next hour. Finally, we also compare the prediction results with LSTM and Transformer models that only consider temporal relationships. The research demonstrates that the Spacetimeformer model consistently captures essential trend changes and provides stable predictions in stock price forecasting. This article proposes a Spacetimeformer model combined with daily moving windows. This method has superior performance in stock price prediction and also demonstrates the significance and value of the space–time mechanism for prediction. We recommend that people who want to predict stock prices or other financial instruments try our proposed method to obtain a better return on investment.

## 1. Introduction

With the progress of society and changes in the economic environment, investment and finance have become mainstream trends in modern society. Among numerous investment options such as bonds, stocks, funds, and futures, stock trading is a popular choice for many people. In stock trading, quantitative trading is an important area that utilizes extensive historical data and market information to predict stock price trends [1,2,3]. Through these predictions, investors can obtain signals about stock price movements earlier and execute buying or selling actions with appropriate timing [4,5]. This behavior is sometimes likened to the concept of insider trading, as investors can act earlier than others based on their predictions to gain better trading opportunities. Therefore, in quantitative trading, how to forecast stock prices is a crucial issue. In recent years, with the development of artificial intelligence, there is an increasing amount of research confirming that neural network models outperform traditional time series methods in stock price prediction [6,7,8,9].

In addition to neural network models, deep learning models are currently the most important models in the field of artificial intelligence, especially in time series forecasting [10,11,12,13]. Among them, the LSTM model [14] is one of the most commonly used deep learning models and has shown outstanding performance in time series forecasting [15,16,17,18,19,20,21,22,23,24]. However, after the Transformer model [25] achieved breakthroughs in the field of natural language processing (NLP), people began to explore whether the Transformer model could also be applied to time series forecasting. The LSTM method was originally developed for NLP, and it was later applied to time series forecasting, which also achieved good results. When Transformer technology was successful in NLP tasks, many data scientists began to apply its technology to time series forecasting, showing the potential of the Transformer model to replace the LSTM model and showing a better performance in time series forecasting [26,27,28,29,30,31,32,33]. Recently, it has become one of the most noteworthy new developments.

Traditional LSTM and recent Transformer models only focus on the temporal relationship between data points, and predict future information by capturing the dependencies between before and after data collection. They overlook the spatial relationships that exist among variables, which can be complex and dynamic in the context of stocks [34,35]. For example, some companies may hold important positions in the Apple’s supply chain at certain times, while other companies may replace these positions at different times. Furthermore, the relationships among these companies are not fixed and may change over time. Therefore, considering the interaction among multiple stocks is crucial for stock price forecasting. However, most advanced models primarily concentrate on the time series characteristics of individual stocks and pay relatively little attention to the interactions among multiple stocks. However, some scholars have proposed methods that integrate spatial and temporal concepts, such as the spatiotemporal attention-based convolutional network (STACN) model introduced by Lin et al. [36]. STACN uses the convolutional neural network to extract spatial feature maps from news headlines to capture the market structure of stocks. Simultaneously, LSTM extracts temporal features from historical stock prices and relevant fundamental information to capture price variations and trends. Finally, STACN employs attention mechanisms to learn and select the most important features, utilizing spatiotemporal information for stock price prediction. Hou et al. [37] also proposed an approach incorporating the graph structure relationships between different companies into the time series forecasting task. Firstly, the method utilizes the Variational Autoencoder (VAE) to learn the low-dimensional latent features from the fundamental data of companies. It further calculates the Euclidean distances between companies to establish a graph network and explore inter-company correlations. Then, a hybrid deep neural network consisting of a graph convolutional network and a long-short term memory network (GCN-LSTM) is used to model the graph structure interaction among stocks and their price fluctuations over time. The method that combines spatial and temporal information for stock price prediction is called VAE-GCN-LSTM by Hou et al. [37]. We can observe that both Lin et al. [36] and Hou et al. [37] conducted research based on the LSTM model and did not utilize the Transformer model.

Based on the research background and motivation mentioned above, this study aims to explore the impact of spatiotemporal relationships on the stock price predictions. To capture the relationships between different stocks at different time points, we employ the Spacetimeformer model [38], which incorporates spatiotemporal mechanisms, as the predictive model for stock prices. Additionally, to avoid the influence of the cross-day prediction, we adopt the daily scan window approach in our experimental design. The main focus of this research is multi-time-step forecasting for the prices of individual stock. In the multi-time-step forecasting task, the Spacetimeformer model predicts the stock prices for every ten minutes within the next hour (six steps in total). Finally, we compare the predictions of the Spacetimeformer model, which integrates spatiotemporal concepts, with the latest Transformer model that purely considers time and the more mature LSTM model in time series forecasting. The goal is to investigate the performance differences between models with spatiotemporal considerations and models that only consider time, as well as to assess the impact of spatiotemporal mechanisms on prediction accuracy.

## 2. Materials and Methods

### 2.1. Datasets

The data for this research come from the stock information of the Taiwan Stock Exchange provided by the stock trading system of Yuanta Securities Co., Ltd. (https://www.yuanta.com.tw/eYuanta/Securities/Stock, accessed on 1 May 2022), which is updated every minute. In order to effectively demonstrate the performance of the Spacetimeformer model, we selected six important constituent stocks from the constituent stocks of the Taiwan 50 Index (as shown in Table 1) announced in June 2022 as our research objects. They are Taiwan Semiconductor Manufacturing Co., Ltd. (TSMC, 2330.TW), United Microelectronics Corporation (UMC, 2303.TW), Delta Electronics, Inc., (DELTA, 2308.TW), Evergreen Marine Corporation (EMC, 2603.TW), Formosa Chemicals & Fibre Corporation (FCFC, 1326.TW), and Yuanta Financial Holding Co., Ltd. (YFH, 2885.TW).

Among these companies, TSMC and UMC are the two leaders in Taiwan’s foundry industry, and they have been able to stay within the top five in the global semiconductor foundry field for a long time. Especially in the 2022 global wafer foundry industry revenue ranking, TSMC ranks first in the world and UMC ranks third in the world. The operating conditions of the two companies affect Taiwan’s economic development and also affect the trend of Taiwan stocks. DELTA is a leading manufacturer of power management and thermal management solutions, and it holds a world-class position in many product fields. It has been included in the Dow Jones Sustainability Indexes for twelve consecutive years (2011–2022). EMC has written many brilliant records in the history of container shipping. So far, it has taken the leading position in the world in terms of fleet size and container carrying capacity. FCFC is one of the main members of the Formosa Plastics Group. Whether it is textile, fiber products or petrochemical products, the company has a leading position in Taiwan and Asia. YFH is a financial holding company that develops on the dual axes of securities investment and commercial banking. Its market share in each business is one of the main market leaders, and it has long been recognized by investors.

**Figure 1:**
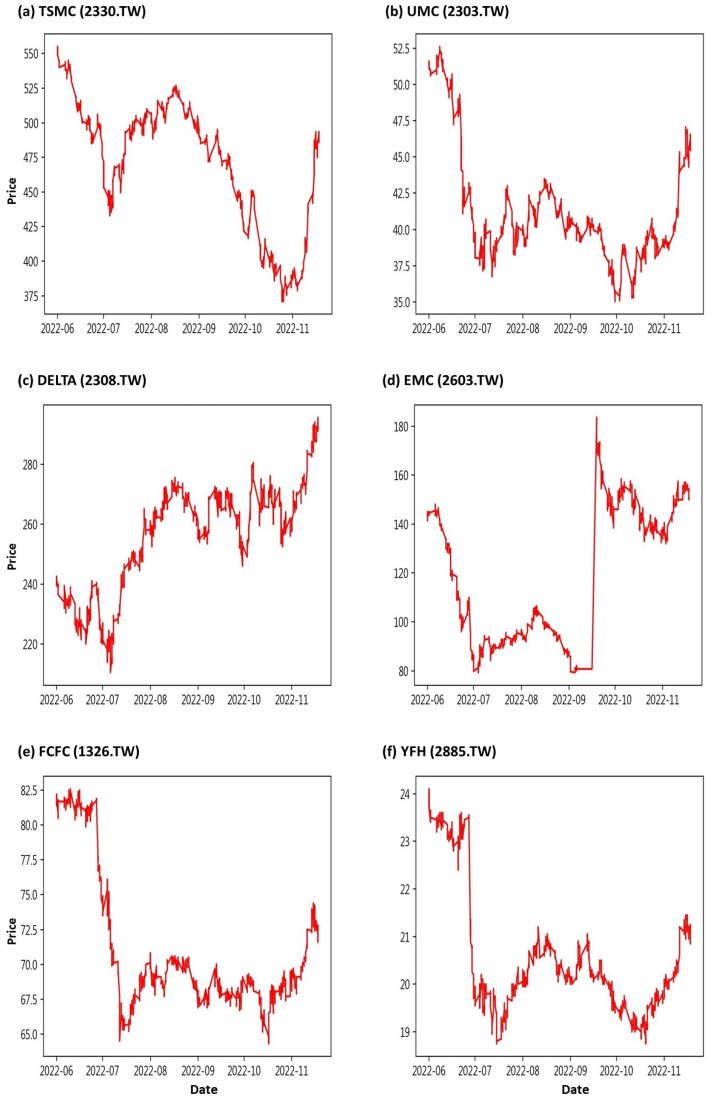
Stock price trend of (**a**) TSMC, (**b**) UMC, (**c**) DELTA, (**d**) EMC, (**e**) FCFC, and (**f**) YFH from 1 June 2022 to 18 November 2022.

We use the ten minute interval trading prices of these six stocks as input data for the model, and the data range is from 1 June 2022 to 18 November 2022. In order to avoid the sharp fluctuations in stock prices caused by “opening” and “closing”, the trading time interval of this study is locked from the original [9:00,13:00] to [9:01,13:21] for discussion. That is to say, we only consider twenty-seven time cut-off points, 9:01, 9:11, …, and 13:21, every day. The descriptive statistics of the stock prices of these six companies are shown in Table 2, where S.D. is the abbreviation of the standard deviation. We can observe that the average stock prices of TSMC, DELTA, and EMC are relatively high, so the stock price variation is also large. The stock price trend chart is shown in Figure 1. We can observe that except for EMC re-listing due to capital reduction on 19 September 2022, the stock price was faulted. The trend of other stocks seems to be very similar to the double bottoms pattern. Therefore, we reasonably suspect that there is some complex relationship among these stocks [39], which we refer to as a spatial correlation.

### 2.2. A Brief Review of LSTM Model

**Figure 2:**
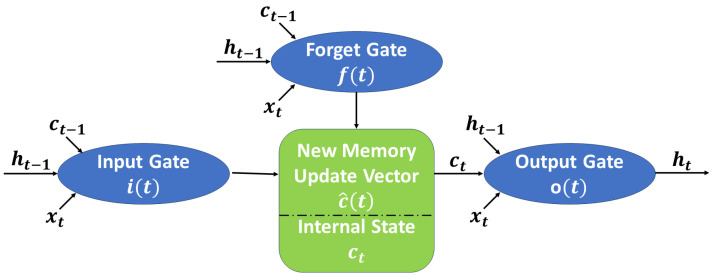
The structure of an LSTM cell.

Predicting stock prices using LSTM models has gained significant attention in financial markets due to their ability to capture complex temporal dependencies in historical price data. LSTM models are a type of recurrent neural network designed to overcome the vanishing gradient problem, making them particularly well suited for time series forecasting. The architecture of an LSTM model is composed of distinct layers, each serving a crucial role in processing sequential data. This design is particularly effective for capturing intricate temporal relationships within the data. A fundamental characteristic of LSTM models is their ability to stack multiple LSTM cells (shown in Figure 2) on top of each other in a layered fashion. This stacking facilitates the hierarchical learning of patterns and relationships within sequential data, making it a robust choice for various time series tasks.

Within each LSTM cell, a series of gates regulates the flow of information throughout the sequence. Their primary function is to control how information is introduced into the network, stored within the cell state, and ultimately released for prediction. The forget gate, in particular, is responsible for filtering out information that the model deems irrelevant or outdated. Only information deemed pertinent and aligned with the model’s learning objectives is retained, while less relevant data are actively discarded. This selective retention and discarding of information through the forget gate enables LSTM models to focus on the most meaningful aspects of the input sequence, enhancing their ability to make accurate predictions and capture underlying patterns effectively.

Let $x_{t}$ be the input at time *t*. We also use $h_{t-1}$ and $c_{t-1}$ to denote the previous hidden state and cell state, respectively, at time (t−1). The initial stage in the LSTM architecture is the forget gate. This gate plays a crucial role in determining the relevance of elements within the cell state, essentially acting as the neural network’s filter for long-term memory. It makes this determination based on information from both the prior hidden state and the new input data. The network operating within the forget gate is trained to assign values close to 0 to information it deems irrelevant and values close to 1 to information it considers relevant. This process allows the LSTM to selectively retain or discard information from the cell state, enabling it to focus on what is most important for its learning objectives. The calculation method of the forgetting probability is given by

(1) $$f\left(t\right)=\mathrm{sigmoid}(W_{f}\cdot \left[x_{t},h_{t-1}\right]+b_{f}),$$

where $W_{f}$ and $b_{f}$ are weight matrix and bias vector parameters, respectively, corresponding to the forget gate which needs to be learned during training.

In the subsequent phase of the LSTM process, we encounter the input gate and the new memory network. Their primary purpose at this stage is to determine what fresh information should be integrated into the network’s long-term memory, known as the cell state. This decision hinges on a careful evaluation of both the previous hidden state and the current input data. Similar to the forget gate, the output value from the input gate holds significant meaning. A low output value signals that the corresponding element of the cell state should remain unaltered, indicating a decision to not update that specific aspect of the memory. Crucially, the new memory update vector serves as a blueprint for adjusting each component of the long-term memory, the cell state. It essentially guides the LSTM in determining how much each memory element should be modified based on the most recent data, ensuring the model’s adaptability and responsiveness to evolving information. Let $W_{i}$, $W_{c}$, $b_{i}$, and $b_{c}$ be the corresponding weight matrices and bias vectors. The functions of the input gate and the new memory network are

(2) $$i\left(t\right)=\mathrm{sigmoid}(W_{i}\cdot \left[x_{t},h_{t-1}\right]+b_{i}),$$

and

(3) $$c\left(t\right)=\tanh(W_{c}\cdot \left[x_{t},h_{t-1}\right]+b_{c}),$$

respectively. The internal state can be updated by

(4) $$c_{t}=i\left(t\right)⊙c\left(t\right)+f\left(t\right)⊙c_{t-1},$$

where ⊙ denotes the Hadamard product.

In the concluding phase of an LSTM’s operation, the pivotal task is to derive the new hidden state by leveraging the recently updated cell state, the preceding hidden state, and the incoming input data. The output gate essentially acts as a decision-maker, regulating which parts of the updated cell state and the previous hidden state should contribute to the final hidden state. This filtration mechanism ensures that only the most pertinent and contextually relevant information is incorporated, enabling the LSTM model to maintain a precise and informative hidden state while avoiding unnecessary complexity. The output gate is calculated as

(5) $$o\left(t\right)=\mathrm{sigmoid}(W_{o}\cdot \left[x_{t},h_{t-1}\right]+b_{o}),$$

where $W_{o}$ and $b_{o}$ are weight matrices and bias vector parameters with respect to the output gate, respectively. The updated cell state is constrained to [−1,1] through the tanh activation function, and then the final new hidden state is given by

(6) $$h_{t}=o_{t}⊙\tanh\left(c_{t}\right).$$

### 2.3. Spacetimeformer Model

Time series forecasting plays an important role in many fields, including weather forecasting, traffic conditions, and financial forecasting. In the past, LSTM had excellent performance in NLP tasks. However, the input of LSTM is a vector, and the input must be processed step by step. Moreover, due to the recursive structure, LSTM cannot capture the long-term correlation in the sequence. Compared with LSTM, the input of the Transformer is a matrix which can eliminate the order of the input so that each Token in the sequence can be processed in parallel. Therefore, the Transformer has been more widely used in time series forecasting tasks recently. A Transformer basically consists of a series of encoder and decoder layers whose input is a matrix. The encoder uses the attention mechanism to understand the correlation between Tokens, while the decoder uses the information obtained from the encoder to produce task-specific predictions.

Time series forecasting is typically based on the sequence-to-sequence approach, where past variable values within a time range of *k* steps are used to predict future target values for *h* steps. We assume that $x_{t}$ represents the timestamp value at time *t* (e.g., year, month, date), and that $y_{t}={\left(y_{t}^{\left(1\right)},y_{t}^{\left(2\right)},\ldots ,y_{t}^{\left(N\right)}\right)}^{\prime }\in R^{N}$, where *N* is the number of variables, represents the response vector at time *t*. Given the timestamps $\left(x_{T-k+1},\ldots ,x_{T}\right)$ and response sequences $\left(y_{T-k+1},\ldots ,y_{T}\right)$, the model would output the response sequences $\left(y_{T+1},\ldots ,y_{T+h}\right)$ for the future steps $\left(x_{T+1},\ldots ,x_{T+h}\right)$. The Informer, proposed by Zhou et al. [33], is an encoder–decoder Transformer architecture for time series forecasting models. This model embeds the time series into a high-dimensional space and uses zeros as placeholders for the unknown target sequence $\left(y_{T-k+1},\ldots ,y_{T},0_{T+1},\ldots ,0_{T+h}\right)$ to embed them into the same dimension. The model adds the timestamps (*x*) and response vectors (y) of the sequence to create an input sequence $Z\in R^{(k+h)\times N}$ consisting of k+h Tokens. This architecture has been demonstrated by Zhou et al. [33] for long-term forecasting. However, this setting will cause the model to learn only temporal features, while ignoring the spatial correlation between response variables.

**Figure 3:**
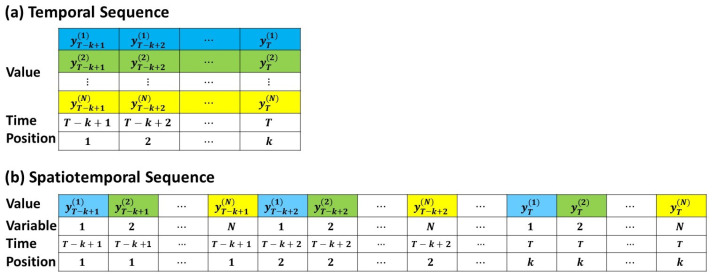
Multivariate time series data. (**a**) Standard temporal input sequence. (**b**) Flattened spatiotemporal input sequence.

In the past, many advanced models for multivariate time series forecasting tasks relied on attention mechanisms between time steps. Such models may be able to capture temporal correlations, but unfortunately have not been able to capture the different spatial relationships between variables. In order to solve this issue, Grigsby et al. [38] proposed the Spacetimeformer model based on the Informer encoder–decoder architecture. The model flattens each multivariate vector ($y_{t}$) along the dimension of time into a timestamped *N* vector to represent the transformation of the input data into a spatiotemporal sequence (shown in Figure 3). Therefore, the new embedded sequence $Z^{\prime }\in R^{N(k+h)\times 1}$ is $(\left(x_{T-k+1},y_{T-k+1}^{\left(1\right)}\right),\ldots ,\left(x_{T-k+1},y_{T-k+1}^{\left(N\right)}\right),\ldots ,\left(x_{T},y_{T}^{\left(1\right)}\right),\ldots ,\left(x_{T},y_{T}^{\left(N\right)}\right),\left(x_{T+1},0_{T+1}^{\left(1\right)}\right),\ldots ,$$\left(x_{T+1},0_{T+1}^{\left(N\right)}\right),\ldots ,\left(x_{T+h},0_{T+h}^{\left(1\right)}\right),\ldots ,\left(x_{T+h},0_{T+h}^{\left(N\right)}\right){)}^{\prime }$. Consequently, when $Z^{\prime }$ passes through the attention layer, there is a direct path between each Token, allowing the model to capture both temporal and spatial information. This enables the Spacetimeformer model to capture correlations between different variables at different time steps.

The order of the inputs cannot be interpreted because the attention mechanism puts the input sequence into the model at the same time. Therefore, we need to add relative position information through position embedding. Time marks are an important feature in time series forecasting; thus, time vector embedding, called Time2Vec embedding [40], is added to capture the periodic and aperiodic relationships of time to produce accurate forecasts. We combine the straightened Time2Vec output with the stock price and project it onto the model through the forward propagation layer, which is called value and time embedding. This is the standard input sequence of the time series forecasting model, which enables each Token to contain time and stock price information. Furthermore, the model also needs to distinguish various stocks at different times; thus, variable embedding is added. The variable embedding straightens the variable indices along the dimension of time and projects each straightened variable index to the same dimension. Finally, the variable values, time embedding, and variable embedding are combined to create the input sequence (shown in Figure 3b) for the encoder, so that each Token carries information about times, stocks, and prices. The attention mechanism is then used to accurately interpret the temporal and spatial information embedded in the sequence.

### 2.4. Experimental Design

**Figure 4:**
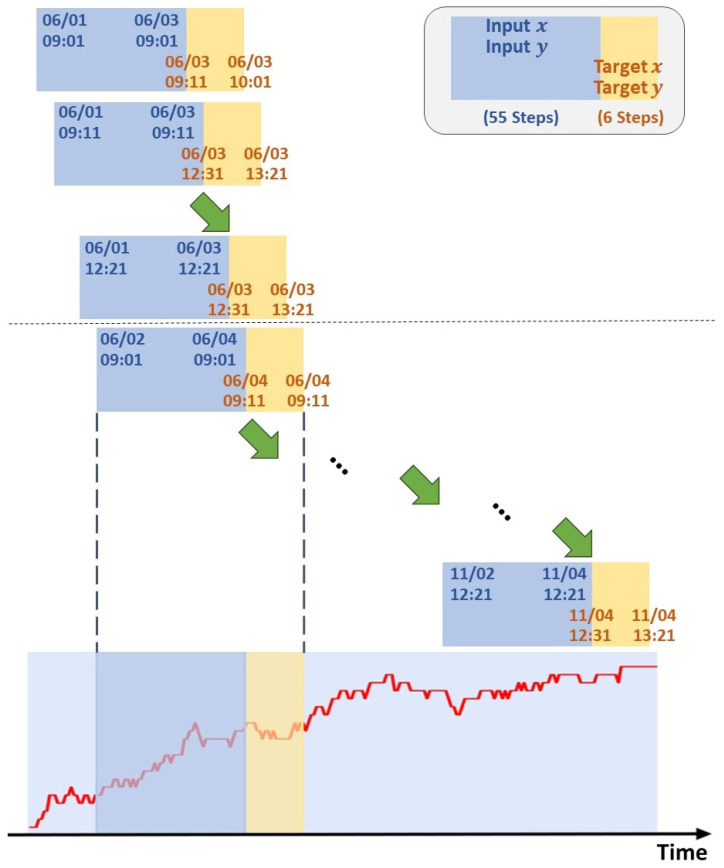
Illustration of the daily moving windows method.

**Figure 5:**
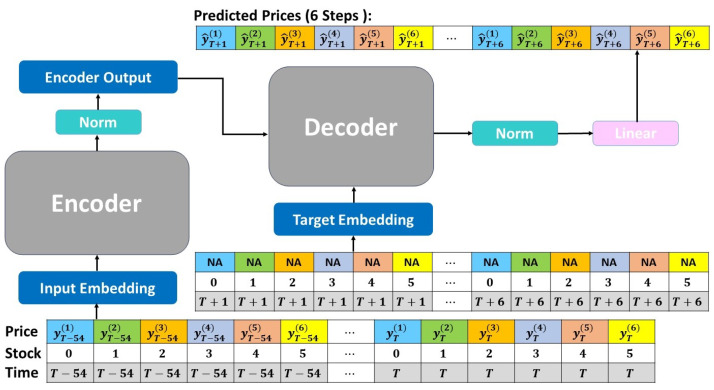
Architecture of the Spacetimeformer model.

In order to avoid affecting the performance of the model due to cross-day forecasting, we use daily moving windows, as shown in Figure 4, to define the training set and testing set. Given any trading day from 3 June 2022 to 4 November 2022, we take time points 9:01, 9:11, …, and 12:21 as starting points. The fifty-five time points forward from this starting point are regarded as the input sequence, and we could predict the stock price (target sequence) every ten minutes in the next hour (a total of six steps). Let us take 9:01 as an example. The model can predict the stock price in the next 6 steps, 9:11, 9:21, …, and 10:01. Until we sample the last time point at 12:21, the model will predict the stock prices at 12:31, 12:41, …, and 13:21. This process, as shown in Figure 5, is repeated until all the training data have been entered into the model, a total of 2205 sequence data. At the same time, we use a total of ten trading days from 7 November 2022 to 18 November 2022 as the testing data.

Finally, we compare the predictions of the Spacetimeformer model, which considers the interaction of time and space, with the Transformer model that only considers temporal correlations. We also explore whether the performance of the above two models is different from that of the earlier LSTM model. We use the mean absolute error rate (MAPE) and root mean square error (RMSE) as the indicators of the performance evaluation among the three models. The formulae of MAPE and RMSE are

(7) $$\mathrm{MAPE}=\frac{1}{n}\sum_{i=1}^{n}\left|\frac{y_{i}-{\hat{y}}_{i}}{y_{i}}\right|,$$

and

(8) $$\mathrm{RMSE}=\sqrt{\frac{1}{n}\sum_{i=1}^{n}{\left(y_{i}-{\hat{y}}_{i}\right)}^{2}},$$

respectively.

## 3. Results

**Figure 6:**
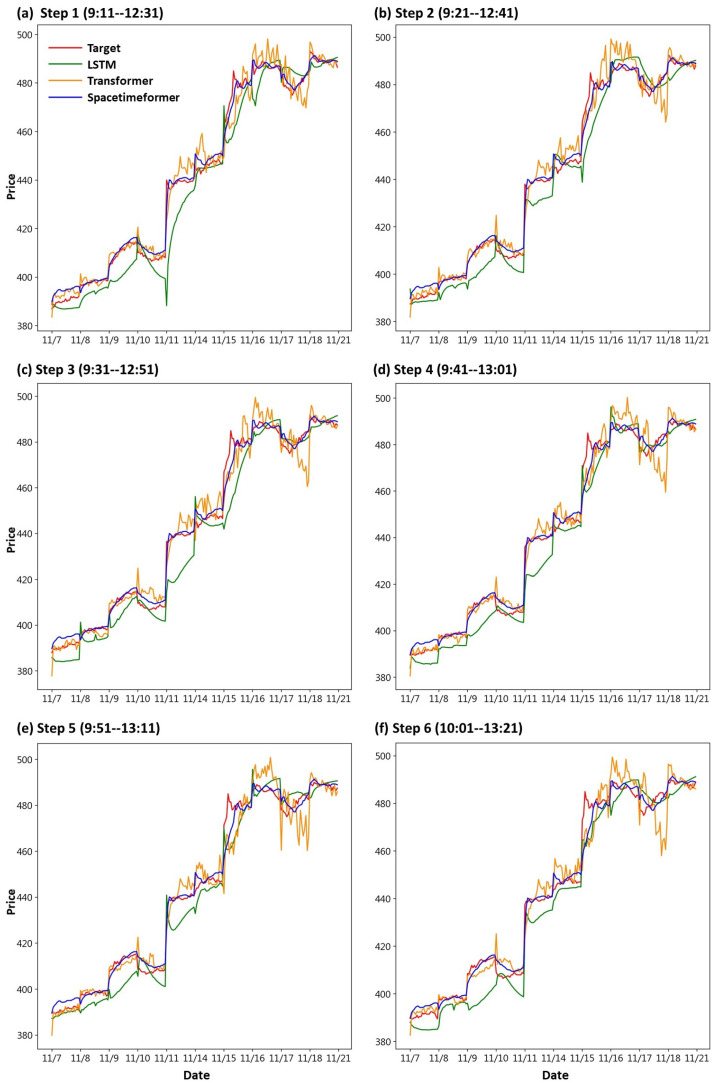
Comparison of the trend chart for TSMC’s stock price using the six-step model forecasting.

**Figure 7:**
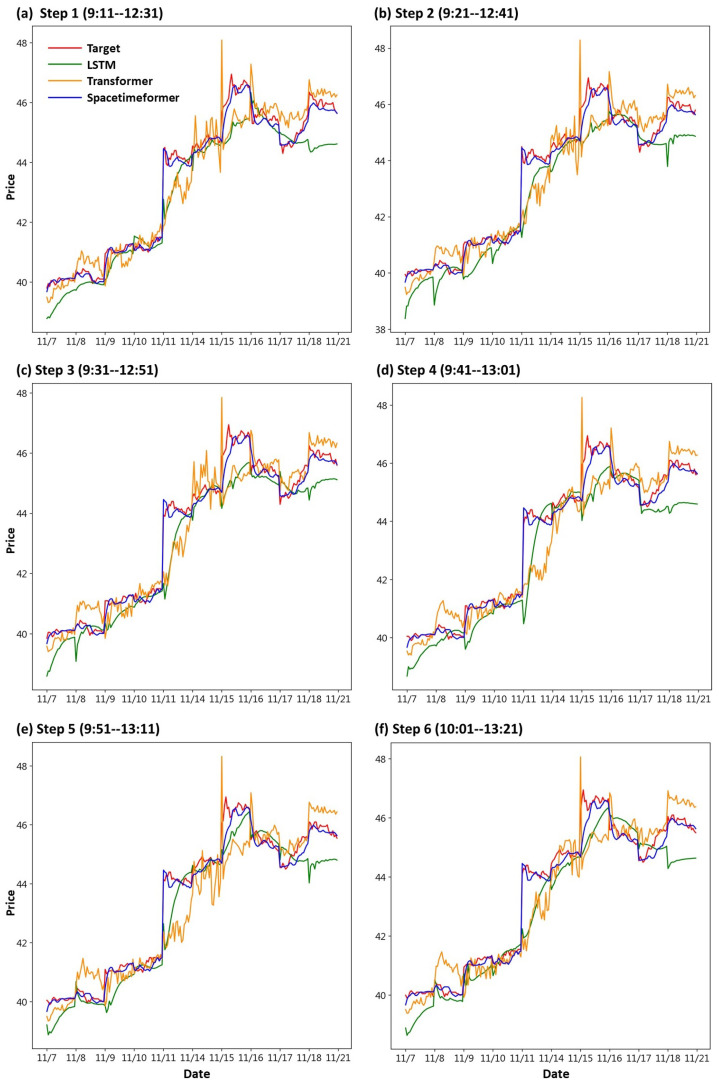
Comparison of the trend chart for UMC’s stock price using the six-step model forecasting.

**Figure 8:**
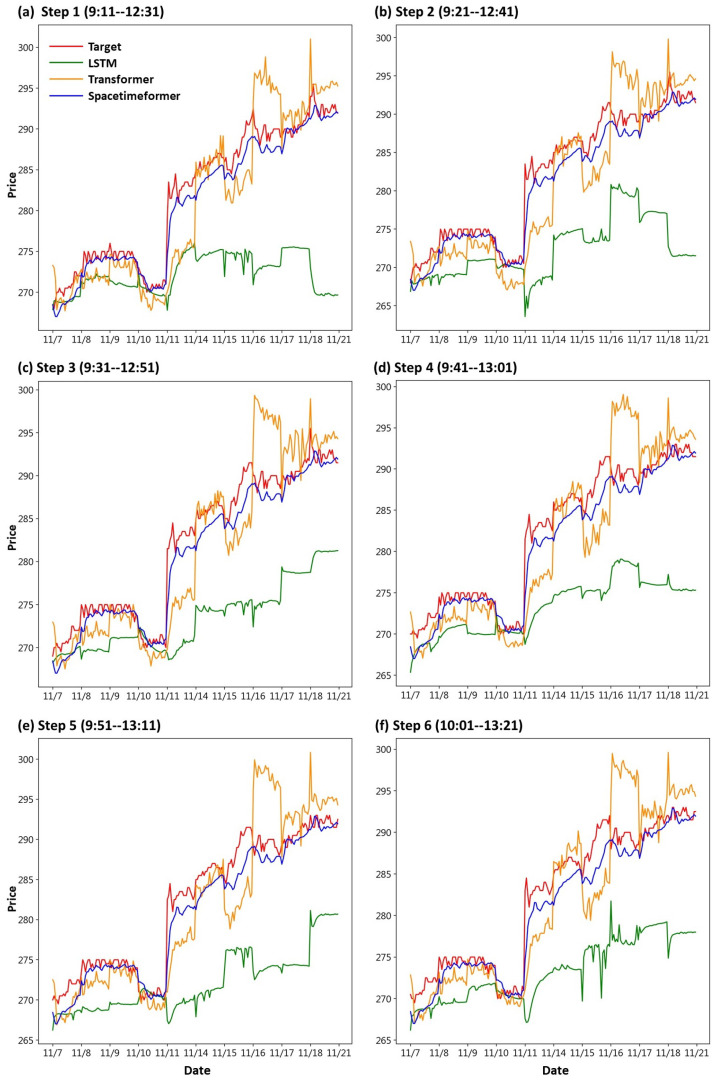
Comparison of the trend chart for DELTA’s stock price using the six-step model forecasting.

**Figure 9:**
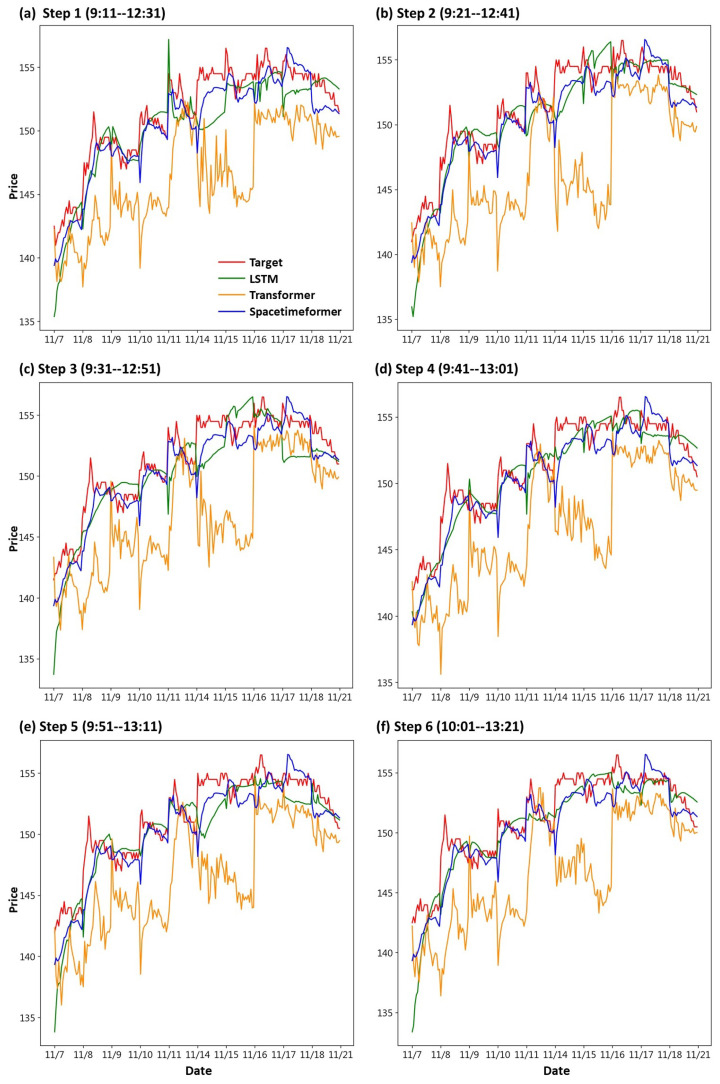
Comparison of the trend chart for EMC’s stock price using the six-step model forecasting.

**Figure 10:**
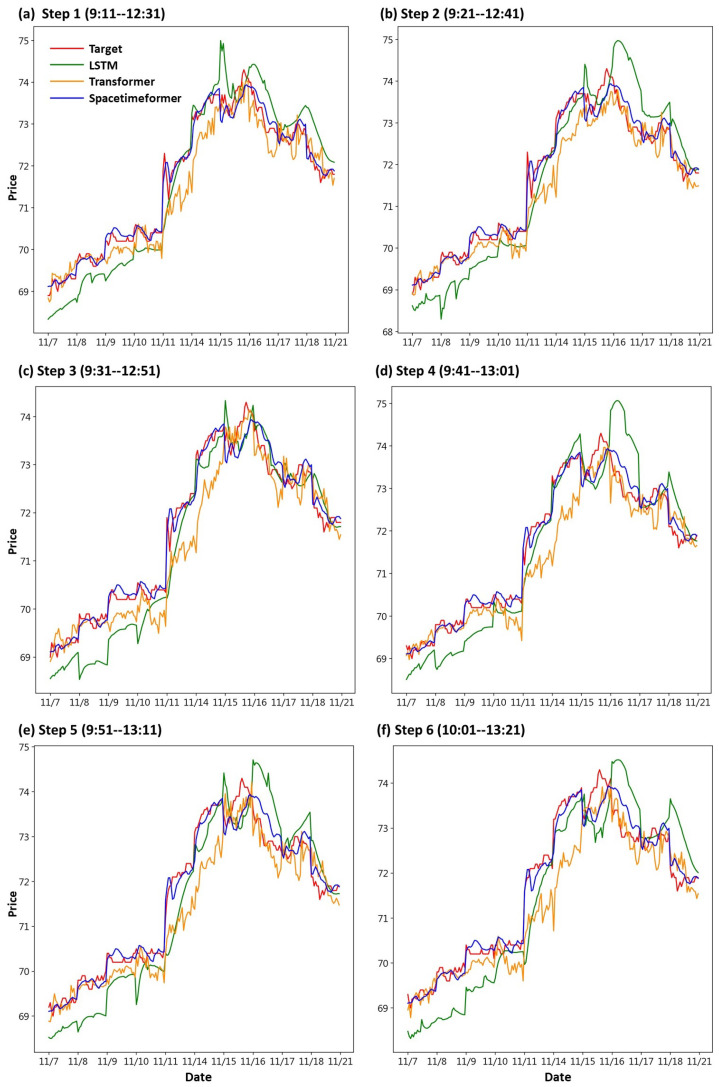
Comparison of the trend chart for FCFC’s stock price using the six-step model forecasting.

**Figure 11:**
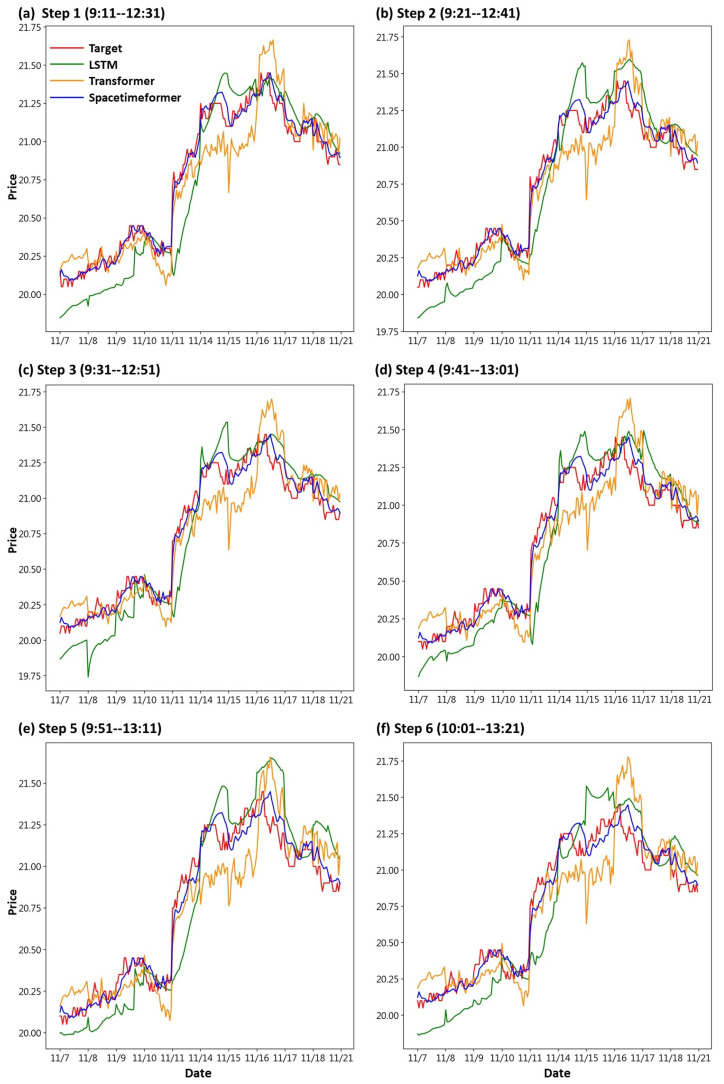
Comparison of the trend chart for YFH’s stock price using the six-step model forecasting.

First, we draw the trend chart of stock price predictions, as shown in Figure 6, Figure 7, Figure 8, Figure 9, Figure 10 and Figure 11, to show the performance results of different models. In all figures, the red line represents the real value, the blue line represents the Spacetimeformer model, the orange line represents the Transformer model, and the green line represents the LSTM model. Among them, “Step *i*”, i = 1, 2, …6, represents the *i*th step predictions through each model. At the same time, we use MAPE (shown in Table 3, Table 4, Table 5, Table 6, Table 7 and Table 8) and RMSE (shown in Table 9, Table 10, Table 11, Table 12, Table 13 and Table 14, respectively) to evaluate the error between the stock price predicted by each model and the real stock price. The trend chart, MAPE, and RMSE help us to more comprehensively evaluate the performance of stock price prediction between models.

Firstly, let us discuss the performance of the stock price forecast for TSMC. From Figure 6, it is evident that the Spacetimeformer model provides the closest predictions to the true stock prices. While the Transformer model exhibits a decent performance in stock price prediction before 16 November, it becomes noticeably distorted thereafter. On the other hand, the LSTM model shows the opposite trend, performing poorly before 16 November and improving afterwards. Furthermore, from the evaluation indexes of MAPE and RMSE in Table 3 and Table 9, respectively, we can observe that the Spacetimeformer model is indeed significantly better than the other two.

For the performance of UMC’s stock price prediction, we can observe from Figure 7 that the Spacetimeformer model maintains robustness. Its forecasted stock price is very close to the actual value. However, the performance of the Transformer model is inferior to the traditional LSTM model. Moreover, from the evaluation indexes of MAPE and RMSE in Table 4 and Table 10, respectively, it is clear that the Spacetimeformer model is better than the other two methods in UMC stock price prediction. However, some predictions of the Transformer model are indeed not as good as the LSTM model.

Next, we discuss the respective predictions of the three models for DELTA’s stock prices. From Figure 8, we can observe that the Spacetimeformer model continues to exhibit the best predictive performance. The Transformer model performed next, but with a significantly larger deviation than previous predictions for TSMC and UMC. On the other hand, the LSTM model performed poorly, with significant bias in stock price predictions. As shown in Table 5 and Table 11, the stock price forecast generated by the Spacetimeformer model is indeed better than the other two models. The predictions of the LSTM model were even more disappointing.

Regarding the stock price forecast of EMC, from Figure 9, we can observe that the stock price forecast of the Spacetimeformer model is still stable. The prediction performance of the LSTM model is the best prediction result so far. The Transformer model performed poorly, with the worst prediction results so far. From Table 6 and Table 12, we can clearly observe that the performance of the Spacetimeformer model and the LSTM model are almost comparable. It is worth mentioning that under the RMSE evaluation standard, LSTM is slightly better than Spacetimeformer in the fourth step of the prediction. However, the stock price prediction of the Transformer model is significantly inferior to the other two.

From Figure 10, we can observe that the Spacetimeformer model’s predictions of TSMC’s stock prices are still better than the other two. The performance of the Transformer model or the LSTM model is still not as good as expected because there is a gap between the prediction and the true stock prices. From Table 7 and Table 13, we can observe that the stock price prediction of the Spacetimeformer model is significantly better than the other two, and the performance of the Transformer model or the LSTM model is poor.

Finally, we discuss the stock price forecast of YFH. From Figure 11, we can observe that the stock price forecast of the Spacetimeformer model is still excellent. However, the Transformer model performs slightly better than the LSTM model, but both often underestimate the stock price. From Table 8 and Table 14, we can clearly observe that the stock price prediction of the Spacetimeformer model is also better than the other two, and the performance of the Transformer model or the LSTM model really needs to be strengthened.

According to the above results, the Spacetimeformer model can predict the stock price in the next ten minutes, twenty minutes or even sixty minutes with the smallest error. It significantly outperforms the other two models for stock price prediction at all steps. The Transformer model is next, and the LSTM model has the highest error. Most of the predictions from the Spacetimeformer model fall close to the true value, despite errors from the true data. However, these errors can be explained as natural fluctuations. Although the Transformer model outperforms the LSTM model, the predictions at some time points are significantly biased. It shows that its smoothness processing is poor, and it also confirms again that the Spacetimeformer model with the concept of time and space performs better in stock price prediction. The Spacetimeformer model can continuously capture important trend changes in stock price forecasts and provide relatively stable forecast results. In the predictions of multiple stock prices, both the Transformer and LSTM models show very unstable performances, which further proves that the Transformer model combined with the daily moving windows method may perform better in long-term forecasting.

## 4. Discussion

Investment and financial management has become an important topic in modern society, and stock trading is an investment project that people have taken much interest in. In stock trading, quantitative trading is a field worthy of research. We can use a large amount of historical data and market information to predict the stock price trend in order to obtain better trading opportunities. In recent years, with the development of artificial intelligence and deep learning, the LSTM and Transformer models have shown good performance in stock price prediction. The traditional LSTM and the latest Transformer models mainly focus on the time-to-time correlation but ignore the spatial relationship between stocks. There are often complex and dynamic relationships between stocks, such as AI concept stocks, electric vehicle concept stocks, etc. It is important to consider the mutual influence between stocks, but LSTM and Transformer models mainly focus on the time series characteristics of individual stocks. The interaction between multiple stocks is relatively less explored. Therefore, this article uses the Spacetimeformer model with a space–time mechanism as a model for predicting stock prices. The model is trained through the interaction mechanism of space and time to capture the relationship between different stocks at different times.

This study uses TSMC, UMC, DELTA, EMC, FCFC, and YFH from the constituent stocks of the Taiwan 50 Index as our research targets. The stock price every ten minutes from 1 June 2022 to 18 November 2022 is used as our research data. At the same time, we avoid the influence of cross-day forecasts, and use the method of daily moving windows to define the training set and testing set. In addition, through multi-time-step forecasting, we can predict the stock prices every ten minutes in the next hour. We compare it with the Transformer and LSTM models that only consider the temporal relationship, and finally use the MAPE index to evaluate the performance of the three models.

The research results show that the Spacetimeformer model with the space–time concept performs better in stock price prediction than the Transformer and LSTM models. The Spacetimeformer model can continuously capture important trend changes in stock price forecasts and provide relatively stable forecast results. Furthermore, the Spacetimeformer model’s predictions for the next ten minutes, twenty minutes, and even one hour are very close to the true values. This highlights the excellent performance of the Spacetimeformer model in both short- and long-term predictions. In contrast, the predictions of the Transformer and LSTM models at different time points might vary, indicating a less stable performance. This also further demonstrates the superior performance of models based on the Spacetimeformer architecture in both short- and long-term predictions. The experimental design of this study adopts a daily moving window approach to avoid the problems of cross-day forecasting and using long-term models for prediction. This allows us to more accurately assess model performance and leverage the adaptive nature of deep learning. In summary, the Spacetimeformer model combined with daily moving windows demonstrates a superior performance in stock price prediction compared to the Transformer and LSTM models, indicating the significance and value of spatiotemporal concepts for predictive modeling.

Therefore, we suggest that people who want to predict stock prices or other financial instruments use the Spacetimeformer model with a time–space interaction mechanism to obtain better results. Based on the above research results, we hope to further verify whether the forecasting performance is also excellent for the stock markets of different countries in the future. We hope to use it to discuss different financial instruments, such as foreign exchange, futures contract, cryptocurrency, etc. This will help us provide more diverse investment forecasts and insights.

## Figures and Tables

**Table 1:** The constituent stocks of the Taiwan 50 Index announced in June 2022.

Stock Symbol									
1101.TW	1216.TW	1301.TW	1303.TW	1326.TW	1590.TW	2002.TW	2207.TW	2303.TW	2308.TW
2317.TW	2327.TW	2330.TW	2357.TW	2379.TW	2382.TW	2395.TW	2408.TW	2409.TW	2412.TW
2454.TW	2603.TW	2609.TW	2615.TW	2801.TW	2880.TW	2881.TW	2882.TW	2883.TW	2884.TW
2885.TW	2886.TW	2887.TW	2891.TW	2892.TW	2912.TW	3008.TW	3034.TW	3037.TW	3045.TW
3711.TW	4904.TW	5871.TW	5876.TW	5880.TW	6415.TW	6505.TW	6770.TW	8046.TW	9910.TW

**Table 2:** Descriptive statistics of stock prices.

Name	Symbol	Mean	S.D.	Minimum	Median	Maximum
TSMC	2330.TW	470.18	45.72	371.00	486.00	555.00
UMC	2303.TW	41.24	3.93	35.05	40.05	52.60
DELTA	2308.TW	255.81	17.97	210.50	260.50	295.50
EMC	2603.TW	117.43	28.17	79.30	106.00	183.50
FCFC	1326.TW	70.92	4.89	64.30	68.90	82.60
YFH	2885.TW	20.53	1.29	18.75	20.15	24.10

**Table 3:** Comparison of the MAPE for TSMC’s stock price from 7 November 2022 to 18 November 2022 using the six-step model forecasting.

	Step 1			Step 2			Step 3		
Date	STF	TF	LSTM	STF	TF	LSTM	STF	TF	LSTM
11/07	0.011	0.008	0.008	0.010	0.006	0.006	0.010	0.006	0.016
11/08	0.002	0.003	0.011	0.002	0.004	0.009	0.002	0.005	0.011
11/09	0.003	0.008	0.023	0.002	0.008	0.024	0.003	0.007	0.015
11/10	0.007	0.008	0.011	0.007	0.009	0.010	0.007	0.015	0.007
11/11	0.004	0.014	0.033	0.003	0.013	0.018	0.003	0.013	0.036
11/14	0.006	0.010	0.004	0.006	0.013	0.006	0.006	0.014	0.008
11/15	0.007	0.022	0.021	0.009	0.020	0.026	0.010	0.021	0.033
11/16	0.002	0.009	0.011	0.003	0.014	0.008	0.004	0.010	0.007
11/17	0.003	0.011	0.011	0.004	0.012	0.009	0.005	0.015	0.006
11/18	0.002	0.005	0.004	0.002	0.004	0.006	0.002	0.005	0.005
Mean	0.005	0.010	0.014	0.005	0.010	0.012	0.005	0.011	0.014
S.D.	0.003	0.005	0.009	0.003	0.005	0.007	0.003	0.005	0.011
	**Step 4**			**Step 5**			**Step 6**		
**Date**	**STF**	**TF**	**LSTM**	**STF**	**TF**	**LSTM**	**STF**	**TF**	**LSTM**
11/07	0.009	0.005	0.012	0.009	0.005	0.004	0.009	0.006	0.014
11/08	0.002	0.003	0.013	0.002	0.003	0.012	0.003	0.003	0.009
11/09	0.003	0.007	0.024	0.003	0.007	0.027	0.004	0.009	0.037
11/10	0.008	0.010	0.006	0.008	0.010	0.010	0.008	0.012	0.011
11/11	0.003	0.011	0.029	0.003	0.012	0.021	0.003	0.012	0.017
11/14	0.006	0.007	0.006	0.006	0.009	0.008	0.006	0.011	0.007
11/15	0.011	0.019	0.019	0.013	0.023	0.019	0.013	0.022	0.017
11/16	0.004	0.013	0.006	0.004	0.015	0.009	0.004	0.012	0.010
11/17	0.005	0.018	0.005	0.006	0.018	0.008	0.006	0.020	0.009
11/18	0.002	0.005	0.004	0.002	0.005	0.004	0.002	0.005	0.006
Mean	0.005	0.010	0.012	0.006	0.011	0.012	0.006	0.011	0.014
S.D.	0.003	0.005	0.009	0.003	0.006	0.007	0.003	0.006	0.009

**Table 4:** Comparison of the MAPE for UMC’s stock price from 7 November 2022 to 18 November 2022 using the six-step model forecasting.

	Step 1			Step 2			Step 3		
Date	STF	TF	LSTM	STF	TF	LSTM	STF	TF	LSTM
11/07	0.002	0.007	0.017	0.002	0.008	0.015	0.002	0.007	0.015
11/08	0.002	0.011	0.007	0.003	0.014	0.009	0.003	0.017	0.007
11/09	0.002	0.010	0.008	0.003	0.008	0.019	0.003	0.010	0.013
11/10	0.002	0.006	0.006	0.003	0.006	0.005	0.003	0.006	0.003
11/11	0.004	0.028	0.018	0.005	0.031	0.023	0.005	0.031	0.024
11/14	0.003	0.010	0.006	0.003	0.009	0.008	0.004	0.011	0.004
11/15	0.006	0.025	0.027	0.008	0.029	0.029	0.008	0.029	0.029
11/16	0.005	0.009	0.006	0.005	0.011	0.003	0.005	0.007	0.008
11/17	0.003	0.016	0.008	0.004	0.013	0.009	0.004	0.010	0.010
11/18	0.004	0.008	0.032	0.004	0.010	0.025	0.004	0.010	0.020
Mean	0.003	0.013	0.014	0.004	0.014	0.015	0.004	0.014	0.013
S.D.	0.002	0.007	0.009	0.002	0.008	0.009	0.002	0.009	0.008
	**Step 4**			**Step 5**			**Step 6**		
**Date**	**STF**	**TF**	**LSTM**	**STF**	**TF**	**LSTM**	**STF**	**TF**	**LSTM**
11/07	0.002	0.007	0.018	0.002	0.008	0.015	0.002	0.008	0.021
11/08	0.003	0.016	0.006	0.003	0.019	0.004	0.003	0.020	0.006
11/09	0.003	0.008	0.017	0.003	0.009	0.017	0.003	0.009	0.011
11/10	0.003	0.005	0.004	0.004	0.005	0.005	0.004	0.006	0.003
11/11	0.005	0.041	0.027	0.004	0.039	0.019	0.004	0.034	0.027
11/14	0.004	0.009	0.004	0.004	0.015	0.004	0.004	0.011	0.010
11/15	0.009	0.030	0.029	0.009	0.031	0.016	0.009	0.030	0.021
11/16	0.005	0.008	0.004	0.005	0.008	0.004	0.004	0.008	0.009
11/17	0.005	0.010	0.014	0.006	0.009	0.011	0.007	0.011	0.009
11/18	0.003	0.011	0.029	0.003	0.014	0.025	0.003	0.016	0.028
Mean	0.004	0.015	0.015	0.004	0.016	0.012	0.004	0.015	0.014
S.D.	0.002	0.011	0.010	0.002	0.010	0.007	0.002	0.009	0.009

**Table 5:** Comparison of the MAPE for DELTA’s stock price from 7 November 2022 to 18 November 2022 using the six-step model forecasting.

	Step 1			Step 2			Step 3		
Date	STF	TF	LSTM	STF	TF	LSTM	STF	TF	LSTM
11/07	0.007	0.007	0.006	0.007	0.007	0.009	0.008	0.007	0.006
11/08	0.003	0.010	0.010	0.003	0.010	0.020	0.003	0.011	0.018
11/09	0.003	0.005	0.013	0.002	0.006	0.013	0.002	0.003	0.012
11/10	0.002	0.007	0.004	0.002	0.010	0.003	0.002	0.006	0.003
11/11	0.008	0.029	0.033	0.009	0.029	0.054	0.009	0.028	0.046
11/14	0.006	0.004	0.039	0.007	0.004	0.040	0.007	0.004	0.041
11/15	0.007	0.016	0.046	0.008	0.020	0.050	0.008	0.017	0.046
11/16	0.007	0.021	0.058	0.006	0.020	0.033	0.006	0.026	0.050
11/17	0.002	0.006	0.051	0.003	0.008	0.046	0.003	0.010	0.040
11/18	0.004	0.010	0.078	0.004	0.007	0.072	0.004	0.007	0.039
Mean	0.005	0.012	0.034	0.005	0.012	0.034	0.005	0.012	0.030
S.D.	0.002	0.008	0.024	0.002	0.008	0.021	0.003	0.009	0.017
	**Step 4**			**Step 5**			**Step 6**		
**Date**	**STF**	**TF**	**LSTM**	**STF**	**TF**	**LSTM**	**STF**	**TF**	**LSTM**
11/07	0.009	0.008	0.011	0.009	0.009	0.011	0.009	0.008	0.010
11/08	0.003	0.010	0.014	0.003	0.009	0.021	0.003	0.009	0.018
11/09	0.002	0.004	0.016	0.002	0.004	0.018	0.002	0.004	0.011
11/10	0.003	0.006	0.018	0.003	0.005	0.002	0.003	0.003	0.002
11/11	0.009	0.025	0.038	0.009	0.023	0.049	0.010	0.022	0.043
11/14	0.007	0.005	0.038	0.007	0.005	0.053	0.007	0.005	0.044
11/15	0.010	0.022	0.047	0.010	0.024	0.044	0.012	0.024	0.048
11/16	0.006	0.027	0.037	0.005	0.030	0.054	0.005	0.028	0.042
11/17	0.003	0.007	0.050	0.004	0.008	0.056	0.004	0.007	0.041
11/18	0.003	0.007	0.057	0.003	0.010	0.040	0.002	0.010	0.050
Mean	0.005	0.012	0.031	0.006	0.013	0.035	0.006	0.012	0.031
S.D.	0.003	0.008	0.018	0.003	0.009	0.019	0.003	0.009	0.017

**Table 6:** Comparison of the MAPE for EMC’s stock price from 7 November 2022 to 18 November 2022 using the six-step model forecasting.

	Step 1			Step 2			Step 3		
Date	STF	TF	LSTM	STF	TF	LSTM	STF	TF	LSTM
11/07	0.010	0.021	0.017	0.010	0.018	0.018	0.011	0.020	0.019
11/08	0.009	0.047	0.011	0.011	0.050	0.010	0.011	0.053	0.013
11/09	0.004	0.026	0.005	0.005	0.027	0.008	0.005	0.022	0.008
11/10	0.006	0.047	0.007	0.006	0.050	0.008	0.005	0.047	0.006
11/11	0.006	0.017	0.009	0.005	0.017	0.010	0.005	0.017	0.013
11/14	0.012	0.051	0.024	0.012	0.057	0.015	0.013	0.055	0.020
11/15	0.007	0.057	0.006	0.007	0.066	0.011	0.007	0.057	0.010
11/16	0.007	0.024	0.007	0.008	0.013	0.005	0.008	0.014	0.003
11/17	0.005	0.024	0.011	0.006	0.013	0.004	0.006	0.011	0.020
11/18	0.010	0.021	0.006	0.009	0.020	0.004	0.008	0.017	0.007
Mean	0.008	0.033	0.010	0.008	0.033	0.009	0.008	0.031	0.012
S.D.	0.002	0.014	0.006	0.003	0.019	0.004	0.003	0.018	0.006
	**Step 4**			**Step 5**			**Step 6**		
**Date**	**STF**	**TF**	**LSTM**	**STF**	**TF**	**LSTM**	**STF**	**TF**	**LSTM**
11/07	0.012	0.023	0.011	0.012	0.030	0.020	0.013	0.026	0.023
11/08	0.012	0.055	0.015	0.013	0.046	0.015	0.014	0.051	0.014
11/09	0.005	0.025	0.005	0.005	0.024	0.004	0.004	0.027	0.005
11/10	0.006	0.050	0.007	0.006	0.051	0.006	0.006	0.051	0.007
11/11	0.004	0.015	0.010	0.004	0.016	0.007	0.004	0.014	0.007
11/14	0.013	0.046	0.010	0.013	0.049	0.021	0.013	0.046	0.011
11/15	0.008	0.056	0.005	0.009	0.057	0.004	0.009	0.056	0.005
11/16	0.007	0.017	0.006	0.007	0.018	0.005	0.007	0.018	0.009
11/17	0.006	0.014	0.006	0.006	0.017	0.012	0.006	0.013	0.003
11/18	0.008	0.017	0.006	0.008	0.018	0.004	0.007	0.014	0.007
Mean	0.008	0.032	0.008	0.008	0.032	0.010	0.008	0.031	0.009
S.D.	0.003	0.017	0.003	0.003	0.016	0.006	0.004	0.017	0.006

**Table 7:** Comparison of the MAPE for FCFC’s stock price from 7 November 2022 to 18 November 2022 using the six-step model forecasting.

	Step 1			Step 2			Step 3		
Date	STF	TF	LSTM	STF	TF	LSTM	STF	TF	LSTM
11/07	0.001	0.004	0.009	0.001	0.003	0.007	0.001	0.003	0.006
11/08	0.001	0.001	0.007	0.002	0.002	0.011	0.002	0.002	0.014
11/09	0.002	0.004	0.009	0.002	0.003	0.008	0.002	0.006	0.009
11/10	0.001	0.004	0.006	0.001	0.004	0.005	0.002	0.006	0.006
11/11	0.002	0.012	0.005	0.003	0.011	0.007	0.003	0.013	0.007
11/14	0.002	0.010	0.002	0.002	0.011	0.004	0.002	0.014	0.004
11/15	0.004	0.005	0.007	0.005	0.007	0.005	0.005	0.004	0.005
11/16	0.003	0.008	0.010	0.004	0.006	0.019	0.005	0.006	0.004
11/17	0.002	0.004	0.005	0.002	0.004	0.007	0.002	0.004	0.003
11/18	0.002	0.005	0.010	0.002	0.005	0.006	0.002	0.005	0.005
Mean	0.002	0.006	0.007	0.002	0.006	0.008	0.003	0.006	0.006
S.D.	0.001	0.003	0.002	0.001	0.003	0.004	0.001	0.004	0.003
	**Step 4**			**Step 5**			**Step 6**		
**Date**	**STF**	**TF**	**LSTM**	**STF**	**TF**	**LSTM**	**STF**	**TF**	**LSTM**
11/07	0.001	0.002	0.006	0.001	0.003	0.008	0.001	0.003	0.011
11/08	0.002	0.002	0.011	0.002	0.002	0.012	0.002	0.002	0.014
11/09	0.002	0.003	0.009	0.002	0.004	0.006	0.002	0.004	0.011
11/10	0.002	0.006	0.004	0.002	0.004	0.005	0.002	0.005	0.003
11/11	0.003	0.013	0.006	0.003	0.014	0.011	0.003	0.014	0.011
11/14	0.002	0.016	0.003	0.002	0.016	0.005	0.002	0.019	0.005
11/15	0.005	0.005	0.007	0.005	0.005	0.007	0.005	0.006	0.010
11/16	0.005	0.006	0.020	0.006	0.006	0.015	0.006	0.006	0.016
11/17	0.003	0.004	0.002	0.003	0.004	0.005	0.003	0.004	0.003
11/18	0.002	0.005	0.007	0.003	0.005	0.005	0.003	0.005	0.013
Mean	0.003	0.006	0.008	0.003	0.006	0.008	0.003	0.007	0.010
S.D.	0.001	0.005	0.005	0.002	0.005	0.003	0.002	0.005	0.004

**Table 8:** Comparison of the MAPE for YFH’s stock price from 7 November 2022 to 18 November 2022 using the six-step model forecasting.

	Step 1			Step 2			Step 3		
Date	STF	TF	LSTM	STF	TF	LSTM	STF	TF	LSTM
11/07	0.002	0.006	0.009	0.002	0.007	0.010	0.001	0.007	0.008
11/08	0.002	0.002	0.010	0.002	0.002	0.010	0.002	0.002	0.013
11/09	0.002	0.004	0.011	0.002	0.005	0.011	0.002	0.005	0.007
11/10	0.002	0.006	0.002	0.002	0.005	0.002	0.002	0.005	0.002
11/11	0.002	0.008	0.018	0.003	0.007	0.011	0.003	0.008	0.014
11/14	0.004	0.012	0.032	0.003	0.010	0.025	0.004	0.010	0.020
11/15	0.002	0.012	0.005	0.002	0.011	0.005	0.003	0.013	0.003
11/16	0.003	0.010	0.004	0.003	0.011	0.011	0.004	0.012	0.006
11/17	0.003	0.004	0.005	0.003	0.004	0.006	0.003	0.004	0.005
11/18	0.002	0.006	0.006	0.003	0.007	0.006	0.003	0.007	0.007
Mean	0.002	0.007	0.010	0.003	0.007	0.010	0.003	0.007	0.009
S.D.	0.001	0.003	0.008	0.001	0.003	0.006	0.001	0.003	0.005
	**Step 4**			**Step 5**			**Step 6**		
**Date**	**STF**	**TF**	**LSTM**	**STF**	**TF**	**LSTM**	**STF**	**TF**	**LSTM**
11/07	0.002	0.008	0.006	0.002	0.007	0.005	0.002	0.008	0.011
11/08	0.002	0.002	0.009	0.002	0.002	0.008	0.002	0.002	0.010
11/09	0.002	0.005	0.008	0.002	0.005	0.010	0.002	0.006	0.012
11/10	0.002	0.005	0.002	0.003	0.005	0.003	0.003	0.005	0.003
11/11	0.004	0.008	0.017	0.004	0.008	0.017	0.005	0.008	0.019
11/14	0.004	0.011	0.029	0.004	0.012	0.025	0.004	0.010	0.028
11/15	0.003	0.009	0.004	0.003	0.014	0.003	0.003	0.014	0.012
11/16	0.005	0.012	0.006	0.005	0.010	0.015	0.006	0.015	0.008
11/17	0.003	0.004	0.010	0.003	0.004	0.005	0.003	0.004	0.004
11/18	0.003	0.007	0.005	0.003	0.009	0.013	0.004	0.008	0.009
Mean	0.003	0.007	0.010	0.003	0.008	0.010	0.003	0.008	0.012
S.D.	0.001	0.003	0.008	0.001	0.004	0.007	0.001	0.004	0.007

**Table 9:** Comparison of the RMSE for TSMC’s stock price from 7 November 2022 to 18 November 2022 using the six-step model forecasting.

	Step 1			Step 2			Step 3		
Date	STF	TF	LSTM	STF	TF	LSTM	STF	TF	LSTM
11/07	4.32	3.69	3.42	4.11	3.09	2.61	3.92	3.42	6.28
11/08	0.94	1.62	4.53	0.96	1.81	3.91	1.07	2.09	4.58
11/09	1.49	3.76	9.40	1.26	3.86	9.76	1.18	3.37	6.50
11/10	2.84	4.07	5.37	2.96	5.11	4.82	3.14	6.75	3.74
11/11	3.07	7.32	18.34	2.62	6.89	8.02	2.24	6.55	16.35
11/14	3.20	5.66	2.24	3.24	6.62	3.39	3.18	7.08	4.07
11/15	4.14	12.72	12.11	5.36	11.79	14.55	6.50	12.00	18.11
11/16	1.68	5.57	6.84	2.10	7.88	4.37	2.05	6.17	3.61
11/17	2.01	6.36	6.02	2.35	7.15	5.35	2.70	8.98	3.22
11/18	1.35	3.08	2.82	1.32	2.80	4.37	1.31	2.80	2.89
Mean	2.50	5.38	7.11	2.63	5.70	6.11	2.73	5.92	6.93
S.D.	1.12	2.92	4.72	1.30	2.83	3.48	1.55	2.93	5.28
	**Step 4**			**Step 5**			**Step 6**		
**Date**	**STF**	**TF**	**LSTM**	**STF**	**TF**	**LSTM**	**STF**	**TF**	**LSTM**
11/07	3.77	2.78	4.96	3.74	2.93	1.63	3.82	3.00	5.76
11/08	1.21	1.50	5.03	1.33	1.26	4.92	1.50	1.32	4.10
11/09	1.36	3.50	10.05	1.60	3.45	11.20	1.97	4.40	15.42
11/10	3.27	5.10	2.66	3.42	5.22	4.66	3.50	6.00	5.42
11/11	2.09	6.03	13.20	2.25	6.29	9.59	2.38	6.40	7.54
11/14	3.21	4.21	3.09	3.26	5.07	4.25	3.02	5.90	3.15
11/15	7.43	11.01	11.46	8.38	14.38	11.59	9.12	12.53	10.26
11/16	2.18	7.05	3.81	2.23	8.15	5.06	2.22	7.14	5.76
11/17	3.01	10.47	2.70	3.38	10.54	4.69	3.67	11.41	5.05
11/18	1.30	3.36	2.57	1.33	2.83	2.80	1.32	3.54	3.60
Mean	2.88	5.50	5.95	3.09	6.01	6.04	3.25	6.16	6.61
S.D.	1.75	3.03	3.84	1.96	3.81	3.30	2.13	3.35	3.53

**Table 10:** Comparison of the RMSE for UMC’s stock price from 7 November 2022 to 18 November 2022 using the six-step model forecasting.

	Step 1			Step 2			Step 3		
Date	STF	TF	LSTM	STF	TF	LSTM	STF	TF	LSTM
11/07	0.08	0.35	0.75	0.10	0.39	0.70	0.09	0.33	0.70
11/08	0.11	0.51	0.33	0.13	0.60	0.52	0.14	0.70	0.38
11/09	0.14	0.50	0.42	0.17	0.45	0.83	0.18	0.52	0.57
11/10	0.11	0.32	0.26	0.13	0.29	0.31	0.16	0.32	0.16
11/11	0.23	1.33	1.06	0.25	1.47	1.31	0.27	1.52	1.43
11/14	0.16	0.54	0.30	0.18	0.50	0.43	0.20	0.59	0.26
11/15	0.39	1.27	1.29	0.47	1.41	1.37	0.51	1.42	1.36
11/16	0.26	0.59	0.33	0.32	0.63	0.17	0.30	0.45	0.36
11/17	0.15	0.79	0.43	0.19	0.68	0.52	0.24	0.52	0.53
11/18	0.24	0.40	1.47	0.22	0.49	1.21	0.20	0.48	0.96
Mean	0.19	0.66	0.66	0.21	0.69	0.73	0.23	0.69	0.67
S.D.	0.09	0.34	0.43	0.10	0.39	0.41	0.11	0.41	0.42
	**Step 4**			**Step 5**			**Step 6**		
**Date**	**STF**	**TF**	**LSTM**	**STF**	**TF**	**LSTM**	**STF**	**TF**	**LSTM**
11/07	0.11	0.35	0.79	0.10	0.37	0.68	0.10	0.37	0.89
11/08	0.15	0.71	0.31	0.15	0.81	0.20	0.14	0.86	0.24
11/09	0.18	0.45	0.77	0.18	0.50	0.78	0.16	0.51	0.48
11/10	0.18	0.27	0.21	0.19	0.26	0.24	0.21	0.30	0.16
11/11	0.26	1.85	1.61	0.23	1.76	1.17	0.20	1.62	1.42
11/14	0.22	0.49	0.23	0.23	0.79	0.22	0.24	0.53	0.54
11/15	0.56	1.46	1.41	0.60	1.48	0.88	0.65	1.45	1.10
11/16	0.27	0.56	0.18	0.26	0.54	0.23	0.23	0.53	0.43
11/17	0.27	0.55	0.73	0.31	0.50	0.56	0.36	0.60	0.46
11/18	0.17	0.54	1.35	0.16	0.66	1.20	0.14	0.75	1.31
Mean	0.24	0.72	0.76	0.24	0.77	0.62	0.24	0.75	0.70
S.D.	0.12	0.49	0.51	0.13	0.46	0.37	0.15	0.42	0.42

**Table 11:** Comparison of the RMSE for DELTA’s stock price from 7 November 2022 to 18 November 2022 using the six-step model forecasting.

	Step 1			Step 2			Step 3		
Date	STF	TF	LSTM	STF	TF	LSTM	STF	TF	LSTM
11/07	1.91	2.10	1.83	2.09	2.15	2.52	2.25	2.18	1.78
11/08	1.04	2.80	2.71	1.18	2.90	5.55	1.21	2.98	4.99
11/09	0.80	1.52	3.72	0.71	1.69	3.59	0.66	1.03	3.34
11/10	0.52	2.20	1.10	0.62	2.98	1.12	0.76	1.73	1.00
11/11	2.74	8.59	9.57	3.13	8.54	15.33	2.96	8.25	13.00
11/14	1.95	1.56	11.24	2.02	1.40	11.52	2.10	1.29	11.67
11/15	2.11	5.38	13.52	2.42	6.38	14.54	2.67	5.66	13.42
11/16	2.09	6.15	16.83	1.96	6.07	9.65	1.93	7.78	14.41
11/17	0.87	2.04	14.80	1.04	2.78	13.36	1.11	3.37	11.75
11/18	1.40	3.18	22.68	1.42	2.58	20.94	1.39	2.47	11.59
Mean	1.54	3.55	9.80	1.66	3.75	9.81	1.70	3.67	8.70
S.D.	0.68	2.25	6.95	0.76	2.26	6.15	0.75	2.50	5.00
	**Step 4**			**Step 5**			**Step 6**		
**Date**	**STF**	**TF**	**LSTM**	**STF**	**TF**	**LSTM**	**STF**	**TF**	**LSTM**
11/07	2.38	2.51	3.03	2.47	2.75	3.13	2.58	2.67	2.89
11/08	1.15	2.84	3.91	1.18	2.60	5.78	1.21	2.53	5.00
11/09	0.70	1.42	4.48	0.70	1.17	4.93	0.64	1.19	2.97
11/10	0.88	1.75	0.63	0.93	1.40	0.71	0.96	1.07	0.74
11/11	3.07	7.42	10.77	3.22	6.85	13.90	3.33	6.59	12.31
11/14	2.20	1.51	10.87	2.18	1.79	15.25	2.26	1.83	12.50
11/15	2.97	6.88	13.66	3.23	7.51	13.04	3.54	7.35	14.20
11/16	1.87	7.93	10.85	1.80	8.72	15.52	1.75	8.18	12.11
11/17	1.15	2.39	14.51	1.17	2.68	16.33	1.20	2.28	11.94
11/18	1.07	2.39	16.80	0.94	3.22	11.82	0.77	3.18	14.56
Mean	1.74	3.71	8.95	1.78	3.87	10.04	1.82	3.69	8.92
S.D.	0.83	2.48	5.24	0.90	2.61	5.50	1.00	2.51	5.07

**Table 12:** Comparison of the RMSE for EMC’s stock price from 7 November 2022 to 18 November 2022 using the six-step model forecasting.

	Step 1			Step 2			Step 3		
Date	STF	TF	LSTM	STF	TF	LSTM	STF	TF	LSTM
11/07	1.56	3.29	2.99	1.62	2.91	3.22	1.81	3.21	3.39
11/08	1.76	7.33	2.13	2.05	7.73	2.02	2.19	7.73	2.28
11/09	0.75	4.05	0.76	0.81	4.10	1.41	0.82	3.52	1.28
11/10	1.51	7.17	1.22	1.50	7.61	1.48	1.36	7.24	1.24
11/11	1.05	3.47	1.61	1.00	3.43	2.08	0.98	3.62	2.43
11/14	2.25	8.03	3.79	2.33	8.91	2.58	2.43	8.70	3.10
11/15	1.28	8.90	1.20	1.25	10.20	1.90	1.23	8.91	1.74
11/16	1.40	3.88	1.46	1.44	2.16	1.00	1.50	2.36	0.67
11/17	0.96	3.77	1.91	1.04	2.06	0.72	1.07	1.91	3.14
11/18	1.73	3.42	1.00	1.62	3.21	0.72	1.52	2.88	1.26
Mean	1.42	5.33	1.81	1.47	5.23	1.71	1.49	5.01	2.05
S.D.	0.42	2.12	0.90	0.45	2.89	0.77	0.50	2.64	0.90
	**Step 4**			**Step 5**			**Step 6**		
**Date**	**STF**	**TF**	**LSTM**	**STF**	**TF**	**LSTM**	**STF**	**TF**	**LSTM**
11/07	1.98	3.57	1.89	2.09	4.55	3.65	2.23	3.93	4.39
11/08	2.39	8.44	2.71	2.53	7.22	2.83	2.74	7.88	2.61
11/09	0.83	3.84	0.82	0.80	3.70	0.77	0.75	4.13	0.93
11/10	1.47	7.69	1.23	1.61	7.81	1.24	1.58	7.72	1.17
11/11	0.94	3.04	1.93	0.83	3.33	1.32	0.69	2.91	1.28
11/14	2.37	7.22	1.74	2.40	7.62	3.37	2.33	7.22	1.76
11/15	1.37	8.71	1.03	1.42	8.79	0.89	1.44	8.68	0.86
11/16	1.42	2.78	1.11	1.51	2.93	1.06	1.54	3.00	1.41
11/17	1.08	2.23	1.03	1.04	2.70	1.83	1.00	2.09	0.69
11/18	1.45	2.90	1.03	1.41	2.98	0.76	1.34	2.43	1.27
Mean	1.53	5.04	1.45	1.56	5.16	1.77	1.56	5.00	1.64
S.D.	0.52	2.49	0.56	0.58	2.28	1.05	0.65	2.44	1.05

**Table 13:** Comparison of the RMSE for FCFC’s stock price from 7 November 2022 to 18 November 2022 using the six-step model forecasting.

	Step 1			Step 2			Step 3		
Date	STF	TF	LSTM	STF	TF	LSTM	STF	TF	LSTM
11/07	0.11	0.32	0.62	0.12	0.25	0.53	0.11	0.27	0.46
11/07	0.11	0.11	0.54	0.13	0.12	0.81	0.14	0.12	0.98
11/07	0.17	0.31	0.67	0.17	0.23	0.56	0.16	0.41	0.67
11/07	0.09	0.31	0.42	0.12	0.37	0.34	0.13	0.51	0.53
11/07	0.24	0.97	0.60	0.30	0.87	0.66	0.28	1.01	0.65
11/07	0.20	0.87	0.20	0.19	0.97	0.31	0.20	1.12	0.31
11/07	0.34	0.46	0.65	0.39	0.65	0.40	0.42	0.40	0.42
11/07	0.26	0.70	0.75	0.32	0.58	1.41	0.37	0.56	0.33
11/07	0.16	0.33	0.36	0.18	0.32	0.50	0.19	0.31	0.26
11/07	0.16	0.46	0.76	0.18	0.41	0.54	0.19	0.43	0.40
Mean	0.18	0.48	0.56	0.21	0.48	0.61	0.22	0.48	0.50
S.D.	0.07	0.26	0.17	0.09	0.27	0.30	0.10	0.27	0.21
	**Step 4**			**Step 5**			**Step 6**		
**Date**	**STF**	**TF**	**LSTM**	**STF**	**TF**	**LSTM**	**STF**	**TF**	**LSTM**
11/07	0.11	0.21	0.44	0.10	0.23	0.58	0.11	0.24	0.76
11/08	0.14	0.13	0.78	0.15	0.16	0.85	0.15	0.13	0.98
11/09	0.16	0.22	0.64	0.16	0.31	0.45	0.17	0.28	0.75
11/10	0.15	0.48	0.27	0.17	0.35	0.47	0.17	0.42	0.25
11/11	0.26	1.01	0.48	0.24	1.09	0.91	0.23	1.10	0.93
11/14	0.21	1.30	0.26	0.19	1.25	0.45	0.22	1.46	0.42
11/15	0.45	0.45	0.59	0.47	0.45	0.63	0.49	0.48	0.83
11/16	0.41	0.51	1.52	0.45	0.54	1.14	0.49	0.52	1.17
11/17	0.21	0.39	0.18	0.22	0.38	0.41	0.22	0.35	0.22
11/18	0.21	0.39	0.68	0.23	0.43	0.49	0.24	0.39	1.08
Mean	0.23	0.52	0.59	0.24	0.51	0.64	0.25	0.52	0.74
S.D.	0.11	0.30	0.37	0.12	0.35	0.23	0.13	0.34	0.32

**Table 14:** Comparison of the RMSE for YFH’s stock price from 7 November 2022 to 18 November 2022 using the six-step model forecasting.

	Step 1			Step 2			Step 3		
Date	STF	TF	LSTM	STF	TF	LSTM	STF	TF	LSTM
11/07	0.04	0.14	0.19	0.04	0.15	0.20	0.04	0.15	0.16
11/08	0.05	0.05	0.20	0.05	0.66	0.20	0.05	0.06	0.27
11/09	0.04	0.10	0.23	0.05	0.11	0.24	0.05	0.12	0.17
11/10	0.04	0.15	0.05	0.05	0.12	0.06	0.05	0.12	0.05
11/11	0.06	0.20	0.40	0.08	0.19	0.27	0.08	0.21	0.32
11/14	0.09	0.26	1.47	0.09	0.23	1.21	0.10	0.23	0.96
11/15	0.05	0.28	0.14	0.06	0.27	0.12	0.07	0.30	0.08
11/16	0.07	0.24	0.09	0.09	0.27	0.25	0.10	0.28	0.15
11/17	0.06	0.09	0.14	0.07	0.09	0.16	0.08	0.11	0.13
11/18	0.06	0.14	0.13	0.07	0.16	0.13	0.07	0.16	0.16
Mean	0.06	0.16	0.30	0.06	0.22	0.28	0.07	0.17	0.25
S.D.	0.01	0.07	0.40	0.02	0.16	0.32	0.02	0.08	0.25
	**Step 4**			**Step 5**			**Step 6**		
**Date**	**STF**	**TF**	**LSTM**	**STF**	**TF**	**LSTM**	**STF**	**TF**	**LSTM**
11/07	0.04	0.16	0.14	0.04	0.14	0.11	0.04	0.16	0.22
11/08	0.05	0.05	0.18	0.05	0.05	0.17	0.05	0.05	0.21
11/09	0.05	0.11	0.17	0.05	0.12	0.21	0.06	0.13	0.26
11/10	0.06	0.13	0.05	0.07	0.13	0.06	0.07	0.12	0.08
11/11	0.09	0.22	0.38	0.10	0.20	0.38	0.11	0.20	0.42
11/14	0.10	0.25	1.35	0.11	0.26	1.20	0.12	0.23	1.31
11/15	0.07	0.24	0.10	0.08	0.32	0.06	0.08	0.33	0.27
11/16	0.12	0.28	0.15	0.12	0.25	0.34	0.13	0.35	0.19
11/17	0.08	0.10	0.24	0.08	0.11	0.13	0.08	0.10	0.11
11/18	0.07	0.18	0.12	0.08	0.20	0.28	0.08	0.18	0.20
Mean	0.07	0.17	0.29	0.08	0.18	0.29	0.08	0.18	0.33
S.D.	0.02	0.07	0.36	0.03	0.08	0.32	0.03	0.09	0.34

## Data Availability

All data for this article can be found at https://www.yuanta.com.tw/eYuanta/Securities/Stock, accessed on 1 May 2022.

## Abbreviations

The following abbreviations are used in this manuscript:

LSTM	Long-Short Term Memory
NLP	Natural Language Processing
STACN	Spatial-Temporal Attention-Based Convolutional Network
VAE	Variational Autoencoder
GCN-LSTM	Graph Convolutional Network and Long-Short Term Memory Network
TSMC	Taiwan Semiconductor Manufacturing Co., Ltd.
UMC	United Microelectronics Corporation
DELTA	Delta Electronics, Inc.
EMC	Evergreen Marine Corporation
FCFC	Formosa Chemicals & Fibre Corporation
YFH	Yuanta Financial Holding Co., Ltd.
S.D.	Standard Deviation
